# Health service behaviors of migrants: A conceptual framework

**DOI:** 10.3389/fpubh.2023.1043135

**Published:** 2023-04-14

**Authors:** Boli Peng, Li Ling

**Affiliations:** ^1^Department of Actuarial Science, School of Insurance, Guangdong University of Finance, Guangzhou, China; ^2^Department of Medical Statistics, School of Public Health, Sun Yat-sen University, Guangzhou, China; ^3^Center for Migrant Health Policy, Sun Yat-sen University, Guangzhou, China

**Keywords:** health service behaviors, migrant, health delivery system characteristics, subgroup-specific factors, conceptual framework

## Abstract

Universal health coverage is vital to the World Health Organization’s (WHO’s) efforts to ensure access to health as a human right. However, it has been reported that migrants, including both international immigrants and internal migrants, underuse health services. Establishing a conceptual framework to facilitate research on the health service behaviors (HSB) of migrants is particularly important. Many theoretical frameworks explaining the general population’s HSB have been published; however, most theoretical frameworks on migrants’ HSB only focus on international immigrants without the inclusion of internal migrants. Of note, internal migrants are much more abundant than immigrants, and this group faces similar barriers to HSB as immigrants do. Based on theoretical frameworks of immigrants’ HSB and Anderson’s behavior model, the author proposes a new conceptual framework of migrants’ HSB that includes both immigrants and internal migrants. The new conceptual framework divides the determinants into macro-structural or contextual factors, health delivery system characteristics, and characteristics of the population at risk and describes subgroup-specific factors. The author added some variables and reclassified variables in some dimensions, including characteristics of health delivery systems and access to healthcare. The characteristics of health delivery systems comprise the volume, organization, quality, and cost of the health delivery system, while the characteristics of access to healthcare include time accessibility, geographic accessibility, and information accessibility. The outcomes of HSB have been expanded, and relationships between them have been reported. The mediating effects of some variables have also been described. This conceptual framework can facilitate a deep and comprehensive understanding of the HSB determination process for migrants, including internal migrants.

## 1. Introduction

“Giving everyone, everywhere an equal chance at a safe and healthy life” is the fundamental object of the World Health Organization (WHO). Therefore, universal health coverage is vital to the WHO’s efforts to ensure the achievement of this fundamental object. The United Nation’s (UN’s) Sustainable Development Goal (SDG) 3.8 also aims to “Achieve universal health coverage, including financial risk protection, access to quality essential health-care services and access to safe, effective, quality and affordable essential medicines and vaccines for all.” (1) However, migrants, defined as “individuals who has changed their usual place of residence, either by crossing an international border (named as international migrants or international immigrants) or moving within their country of origin to another region, district or municipality (named as internal migrants (IMs))” (2), are often excluded from the local health systems and tend to underuse the available health services (3, 4). Exploring the health service behaviors (HSB) of migrants could promote universal health coverage and contribute to the achievement of the SDG. Establishing a conceptual framework to facilitate research about migrants’ HSB is particularly important.

Many theoretical frameworks for explaining the general populations’ HSB have been published. However, theoretical frameworks for migrants were insufficient. Few theoretical frameworks accounted for the IMs. The 2022 World Migration Report reported 281 million international immigrants, while the most recent estimate of IMs globally was 740 million in 2009, which is over three times the number of international immigrants at the time (2, 5). Much larger numbers migrate within their own countries (5). Similar to international immigrants, the IMs always suffer from poor living (6) and working conditions (7, 8) and unequal access to various social welfare programs (9), including health services (10–12), than the local residents. Consequently, IMs had various barriers to accessing health services similar to immigrants. Previous studies on IMs’ HSB always reported the results based on immigrants. To some extent, the theoretical framework for explaining immigrants’ HSB would also be appropriate for IMs.

However, migrating within their home countries, IMs would suffer fewer language and/or communication barriers, cultural differences, and information barriers that would be encountered in a new health system. Some characteristics of migration, such as the reason for migration and migration range (divided into migration across provinces, cities, or counties/districts in China), were more prominent as the determinants of IMs’ HSB (13–15). According to the Seventh National Population Census, there were 261 million IMs in China in 2020, making up the majority of IMs worldwide. Although the majority of them in China enrolled in social medical insurance, they might encounter difficulties in using them in local places due to the bad flexibility caused by conflicts between cross-regional health service utilization (HSU) and localized and fragmented management of medical insurance (16, 17). Suffering from different challenges, the IMs and immigrants would have different principal reasons for opposing HSB. In other words, some determinants might be the main barriers to immigrants’ HSB, but they hardly affect IMs’ choices in terms of HSB. Considering these differences, it is important to clarify the determinants of HSB between immigrants and IMs in the theoretical framework of migrants’ HSB.

In addition, IMs can choose either the host cities for health services or their hometowns where their medical insurance always belongs (18, 19). Medical services in different locations have various expenses and thus could affect the expenditures for medical care in migrants’ hometowns (20), especially when the medical expenses are paid by the hometown’s medical insurance. Unpredictably high expenses outside the regions could result in large insurance payments. In terms of immigrants, medical expenses and financial burdens for health services also differ across countries (21). Consequently, medical location is an important characteristic of HSB. Moreover, the medical location was found to be associated with other characteristics of HSB, such as the site of medical service (22, 23).

This study aimed to explain the HSB of migrants comprehensively by establishing a conceptual framework based on the theoretical framework of the general population and immigrants. Establishing the conceptual framework of migrants’ HSB would yield information concerning several aspects of migrant-related HSB: (1) to help further distinguish the determinants of immigrants’ and IMs’ HSB; (2) to gain new knowledge on the determinants of immigrants’ and IMs’ HSB; (3) to explain the relationships between different characteristics of HSB and differences of their determinants; and (4) to help manage medical insurance funds.

## 2. Existing theoretical models of health service behaviors

Many theoretical models of HSB are described in the literature. As concluded by Yang and Hwang (24), the models proposed before the 1990s explain people’s HSB through illness and stages of illness behavior or medical care (named sociological models), psychological factors or processes (named socio-psychological models), the structure of the healthcare system (named institutional models), and health belief models. All of these models focus on individuals or different aspects of the healthcare institution but neglect other factors related to the health service delivery system and macro-structural or contextual factors.

### 2.1. Anderson’s health behavior model

The most famous model explaining people’s HSB and used in most studies in the literature is Anderson’s health behavior model (hereinafter referred to as Anderson’s model). Anderson’s model was first proposed in 1968 and revised three times afterward (25). The initial model explained people’s HSU based on three sets of factors: (1) predisposing characteristics (demographic, social structure, and health beliefs); (2) enabling resources (personal/family and community); and (3) need (perceived and evaluated). The mediating relationships occurred systematically in sequence. The phase two model added factors of the healthcare system that included policy, resources, and organization. In addition, the model in this phase specified the type, site, purpose, and time interval of HSB. The mediating relationship occurred between the population characteristics and the healthcare system. In phase three, a new category of the determinant of health behaviors was added, namely the external environment. The outcome was also expanded to HSU and personal health practices. The phase four model had the most complex form. The determinants of health behaviors consisted of the healthcare system, external environment, and population characteristics mentioned in the initial model; the former two categories of factors had mediating effects on the population characteristics. The outcome had a feedback effect on population characteristics (25).

To predict HSB, the models proposed before phase four might be more effective, namely the initial model with the addition of the healthcare system, environmental factors, and specified dimensions of the HSB. However, Anderson’s model was proposed to explain general populations’ HSB. Migrants differ from the general populations in social welfare, living, and working conditions as mentioned earlier, thus the migrant-specific factors need to be distinguished. The original model also needs to be revised as new determinants are discovered.

### 2.2. Immigrants’ health behavior model

Based on Anderson’s models and literature on immigrant’ HSB, several theoretical frameworks on immigrant-related health service utilization were proposed in previous studies. Helena Legido-Quigley and colleagues (26) examined the barriers that immigrants suffered in accessing healthcare and categorized these factors into leadership/governance, healthcare financing, service delivery, health workforce, information and research, medical products and technologies, and migrants’ healthcare-seeking behavior. Helena Legido-Quigley and colleagues highlighted environmental factors, factors of the healthcare system, and population characteristics without indicating mediating relationships that might have occurred among them.

Yang and Hwang’s (24) immigrant health behavior model comprehensively describes the determination factors of immigrants’ HSU. They divided the determinations into macro-structural or contextual factors and characteristics of the population at risk (consisting of predisposing factors, enabling resources, and needs), which is consistent with Anderson’s model. Referring to the determinants, Yang and Hwang considered the influence of non-health policies and divided resources into three types: (1) financial resources; (2) social resources; and (3) access to healthcare. They further categorized immigrant-specific factors: (1) immigrant-specific health needs or conditions; (2) homeland-based financial and social resources and transnational access to healthcare; (3) immigrant-specific predisposing factors; and (4) context of emigration, reception, and HSU in the homeland. They refuted the overall mediating relationships occurring and considered the mediating effects of some specific determinants and addressed those specific influences.

Yang and Hwang’s immigrant health behavior model was proposed based on Anderson’s model and followed the classification of determinants in this model. However, Yang and Hwang ignore the influence of the healthcare delivery system, which was another important component of HSB, whereas Helena Legido-Quigley and colleagues highlight the influence of the healthcare delivery system. These specific factors of the health delivery systems in the latter model would help to improve Yang and Hwang’s immigrant health behavior models.

All these models excluded IMs, a much larger group. IMs suffer from bad socioeconomic status and less social welfare and thus have more barriers to health services. IMs also differed from immigrants in many aspects as mentioned earlier. Therefore, it is necessary to revise the existing models and distinguish the determinants of HSB between the two subgroups.

## 3. Conceptual framework of this study

**Figure 1:**
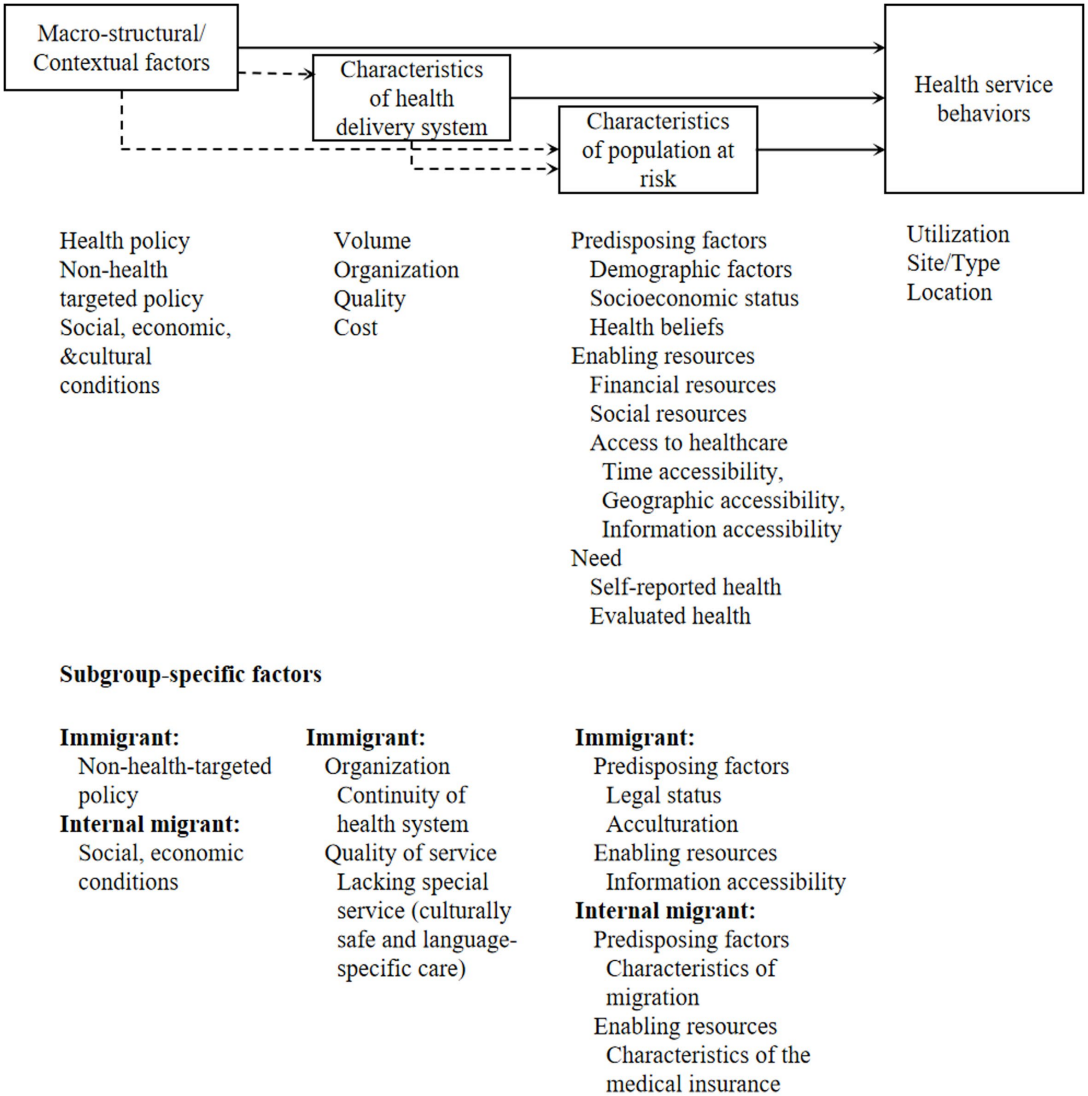
An analytical framework for migrants' health service behaviors. A solid line denotes a direct effect; a broken line indicates that some of the factors within this category have an indirect effect on the outcomes of migrants' health service behaviors.

Based on Anderson’s (25) and Yang and Hwang’s immigrant health behavior models (24), we constructed the fundamental framework of this study. We searched literature published between January 2010 and January 2022 in the following electronic databases: Science Direct, Web of Science, PubMed, Wiley Online Library, and Google Scholar. We also conducted an additional search using the snowballing method. Search terms associated with “migration” or “immigrant” and “health service,” “health facilities” or “medical location” were applied. Combining determinants mentioned by Helena Legido-Quigley and colleagues (26) and reviewing the literature on migrants’ health service utilization, choice of health facilities, and medical locations, the authors supplemented and revised the existing framework of Yang and Hwang’s immigrant health behavior models (24) and proposed the conceptual framework of this study (Figure 1) based on the grounded theory. Specifically, according to the previous studies, we supplemented the dimension of health delivery system characteristics, expanded the health service behaviors, health delivery system characteristics, and access to healthcare, and distinguished determinants of immigrants’ and IMs’ HSB. However, we did not emphasize the immigrant-specific factors mentioned in Yang and Hwang’s immigrant health behavior models except for the immigrant-specific predisposing factors because this study focused on distinguishing subgroup-specific factors between immigrants and IMs. The other immigrant-specific factors on macro-structural/contextual factors, enabling resources, and needs were integrated into the general factors if they worked similarly in the two subgroups.

### 3.1. Health service behavior

#### 3.1.1. Definitions and measurements

Since the dimensions of HSB reflect different aspects of health service-seeking behavior and the determining factors and their mechanism are not the same as each other, it is necessary to specify these dimensions. According to the definition in Anderson’s HSB model (27), we updated the characteristics of HSB and included utilization, type, and site. The utilization of HSB was divided into use or no use.

The updated type refers to the kind of service received and the provider (27) by merging the type and purpose in Anderson’s model: (1) preventive service; (2) outpatient service; (3) inpatient service; (4) emergency services; (5) dental service; (6) pharmacist service; (7) traditional health services; and (8) others. These types of illness-related services (except preventive services) can be substituted for each other up to a point.

Another characteristic of HSB is the site, which differed from the site mentioned by Anderson; it refers to the place (types of health institution) at which the care was received: (1) clinic; (2) hospital; or (3) other. Clinics and hospitals include private and public institutions. The classification of the site can vary among different countries according to the structure of their healthcare system. For instance, in some countries (such as China), the health delivery system has different levels of healthcare institutions (28–32). All healthcare institutions provide similar illness-related services. A higher level of healthcare institution incorporates more experienced experts and advanced equipment. Patients are permitted to choose the health facility (28–31) and tend to prefer doctors at higher-level healthcare institutions (33). The site of HSB in these countries may be further divided into primary, secondary, and tertiary health institutions.

#### 3.1.2. Migrant-specific characteristics of HSB

Migrants who migrate from their hometown to another destination can use health services in both places; hence, their HSU can also be characteristic of a specific location. The location of HSB refers to the location at which the care was received, namely hometown or home country, host city or the destination, or other places.

#### 3.1.3. Interaction effect between characteristics of HSB

Characteristics of the healthcare site were found to be associated with location (22). Migrants may return to use health services of higher healthcare facilities, which are generally regarded as higher quality (23). Some literature also found that migrants may return for services in lower-level health facilities as required by the health policy, such as the hierarchical medical system in China (22). Migrants also tended to seek healthcare services in high-level hospitals with higher perceived quality in locations other than their hometowns or destinations (22), which was consistent with the purpose of transnational medical tourism.

Characteristics of type and site are closely associated with each other. The type was more frequently mentioned in literature on HSB of immigrants, while the site was more important to IMs, especially those living in countries with multi-level health providers but less restriction in terms of choosing them. Both types and sites were associated with healthcare accessibility and indicated the quality of service that migrants receive.

### 3.2. Health service utilization of migrants

In general, immigrants (3, 34–36) and IMs (12, 37, 38) have poor HSU levels when compared with the local residents. Factors associated with migrants’ HSU could be divided into three dimensions: (1) macro-structural or contextual factors; (2) characteristics of the health delivery system; and (3) characteristics of the population at risk (24, 27).

#### 3.2.1. Macro-structural/contextual factors and HSU

According to Yang and Hwang (24), macro-structural or contextual factors refer to those that were at the societal or community level and beyond individual control. Macro-structural and contextual factors could be divided into healthcare policy, non-health-targeted policy, and specific regional context factors related to larger social, economic, and cultural conditions (24).

##### 3.2.1.1. General factors

Healthcare policy was found to be an important determinant of both immigrants’ and IMs’ HSU because legislation is always not migrant-inclusive in the healthcare policies. Legal status is always considered one of the most important determinants of immigrants’ health service access. One such example is South Africa; although the South African Constitution and the National Health Act mandate universal health coverage, the Immigration Act and the 2019 NHI Bill demands that people with a legal immigrant status be able to access healthcare (39). Immigrants in host countries generally suffer from complicated bureaucratic policies (40, 41) and specific legal barriers (42, 43). Specifically, the registration procedure regulations (44), inability to verify their identities (45), legal barriers in accessing healthcare (3), and/or lack of confidence in the legal system (46) consequently limit immigrants’ HSU. Similarly, cumbersome administrative procedures and a lack of specific strategies for IMs’ health access also exclude IMs from certain medical services (47). IMs in China also have limited access to health services in their local residence if they do not register with the local government or have stable work in the host city. Residence permits aimed to eliminate inequity rights between IMs and local residents have limited effect on facilitating IMs’ health service access in megacities in China (4). The on-the-spot medical bill settlement system in China aimed to achieve portability of medical insurance and thus was expected to have a positive effect on IMs’ HSU; however, the policy’s effect has yet to be studied.

##### 3.2.1.2. Subgroup-specific factors

Internal migrants’ HSU was only influenced by healthcare policies, while immigrants’ HSU can also be affected by non-healthcare policies. Juárez et al. found that non-health-targeted policies, such as restricted eligibility for welfare support, would decrease HSU without reducing public health insurance coverage (48). The threat of deportation also presents a barrier to health service access (43, 49).

Specific regional context factors related to greater social, economic, and cultural conditions also make sense. Referring to IMs, the specific regional context would include geographical location (50), urban/rural areas (51–53), economic development level of the region (54), city service quality index (55, 56), community health index (55), and/or the proportion of ethnic minorities (56), in addition to cultural factors (47, 57, 58). These aspects are usually associated with the health service resources, quality and accessibility of the service, financial resources, and cultural comfort during HSU. Studies concerning immigrants mainly cite cultural aspects, such as supportive communities (49) and/or immigrant-friendly environments (49), sociocultural structure and deep stigma about a certain illness (such as Chagas Disease (59)), racialized medical perceptions (42), and gender-based cultural norms, including information barriers and stigma (42). In a word, factors of regional context appeared to be more complex among IMs, while the literature on immigrants mainly focused on the cultural aspect because cultural difference between countries was more significant, and health beliefs and habits would differ across countries.

#### 3.2.2. Characteristics of health delivery system and HSU

Characteristics of health delivery systems could be categorized into the volume, organization, quality, and cost of the healthcare delivery system.

##### 3.2.2.1. General factors

First, the volume of the healthcare delivery system was associated with migrants’ HSU because the capacity of the healthcare delivery system is reflected through its volume. A specific healthcare delivery system would first meet the healthcare needs of local residents. Limited healthcare resources (60) and an overburdened public health system (61) were found to be barriers to immigrants’ HSU. IMs’ HSU was also limited by the volume of medical institutions (47, 56) and hospital beds (56).

Second, according to the law of supply and demand, the cost is another characteristic related to migrants’ HSU. Healthcare costs, which are always prohibitive (43), were felt to be one of the main barriers to healthcare access by both immigrants (44, 60, 62–65) and IMs (66–68). Although at times the treatment costs were not considered as barriers, co-payment by patients (69) and the unpredictable nature of out-of-pocket expenses (70) would also limit immigrants’ and IMs’ HSU. In addition to the high cost of healthcare services, difficulty meeting the costs of transport to appointments (65) was also reported.

Third, the healthcare system’s organization influences migrants’ HSU because the organization determines the access, progress, results, and efficiency of the healthcare service. Accessibility of the healthcare system was frequently mentioned in the literature in terms of both immigrants (44, 65, 71) and IMs (58, 72, 73) and measured in different ways. For example, immigrants in host countries always have difficulty navigating a new healthcare system (44, 71). The healthcare system of India restricts some treatment to Indian nationals (45). The limited availability of female-only safe spaces has led to a reduction in refugees’ (61) and IMs’ (58) utilization of maternity care. Furthermore, the efficiency of the healthcare system has also been reported in many literature studies. Long waiting times were considered the main barrier for the immigrants (64, 69, 74) and IMs (68), while the time consumed by medical treatment (67) would also affect the HSU of IMs.

Fourth, quality of the service (75) was reported to be one of the main factors associated with immigrants’ HSU, but reports concerning this aspect in terms of IMs are seldom found in the literature. The reason for this might be a lack of information. Subjective quality of service was important because better subjective satisfaction would increase the desire to seek healthcare when needed. The perceived poor quality of care (43), low standard of care in private clinics (60), or fear of harmful/poor treatment (44) were cited as barriers to immigrants’ and IMs’ (73) HSU. Some literature also refers to the objective quality of service, such as premature hospital discharge (74). Moreover, experiences or relationships with health personnel (71, 76) and trust in health providers (46, 77), factors that are related to the quality of service, were also cited as influencing factors of immigrants’ and IMs’ (58, 73, 78) HSU.

##### 3.2.2.2. Subgroup-specific factors

First, the organization of the healthcare system was described in different aspects between immigrants and IMs, and the continuity of the healthcare system was mentioned only in the literature concerning immigrants. Fragmentation of the healthcare system (60), lack of continuity of care (46, 79, 80), and not having a general practitioner (81) were frequent complaints expressed by immigrants with unmet healthcare service needs. This difference might be because the structure of the healthcare delivery system and standard for healthcare services in different regions of a certain country are similar to each other.

Second, some factors of quality of the service were immigrant-specific factors, namely discrimination (39, 82, 83) and difficulty in communication (80) due to cultural differences (61, 84) and language barriers (74) during healthcare service. These factors were also important determinants that limited immigrants’ HSU because they determine the affective comfort during the service. Consequently, lacking special services, such as culturally safe and language-specific care (71), was also found to be associated with immigrants’ HSU.

##### 3.2.2.3. Mediating effects on the relationship between macro-structural/contextual factors and HSU

Healthcare delivery systems in terms of volume, organization, quality, and cost in most countries are mainly run and funded by the governments or at least managed by the government (28, 29, 32, 85) and thus are influenced by government policy (86). Policies issued by the government can determine resource distribution, organization, financing, and pricing of the healthcare delivery system. Health providers’ attitudes toward immigrants were also found to be associated with the social context of an immigrant-friendly environment.

#### 3.2.3. Characteristics of the population at risk and HSU

Characteristics of populations at risk associated with migrants’ HSU are related to the predisposing factors, enabling resources, and needs (27).

##### 3.2.3.1. General factors

###### 3.2.3.1.1. Predisposing factors

Predisposing factors associated with HSU are constituted by demographic characteristics, socioeconomic status, and health beliefs (27).

Referring to demographic characteristics, general factors, including gender, age, marital status, and education level, were found to be associated with health knowledge and attitude and thus affected migrants’ HSU. Gender was found to be associated with both immigrants’ (64, 71, 87–91) and IMs’ (12, 13, 17, 50, 55, 92–94) HSU; however, the association was mixed. Age also had a mixed effect on immigrants’ (64, 90) and IMs’ (12–14, 38, 92, 95, 96) HSU. Marital status was more frequently regarded as a protective factor for immigrants’ (81, 89, 97) and IMs’ (13, 14, 17, 50, 55) HSU, while some articles reported that marital status had no influence on HSU by IMs (12, 38). Higher education level was also shown to play a protective role in both immigrants’ (98) and IMs’ (99–104) HSU but did not have any influence on HSU in a few articles about IMs (38). Furthermore, IMs’ HSU is associated with the type of *hukou* (100, 105). The *hukou* system is a household registration system in China, categorizing people as either rural or urban residents (106). People with different *hukou* had different living environments, habits, and health beliefs and thus migrants from urban or rural areas had different HSU.

Migrant-specific demographic factors, including generation, race or ethnicity, and duration at a specific destination, were associated with immigrants’ and IMs’ HSU. First-generation immigrants (64, 107–109) and old-generation IMs (50) had worse HSU than the others because the early generation had more barriers to becoming integrated into society. The home country of the immigrants, including Asia or developing countries (88–91, 110, 111), and nationality (112) of IMs were found to be associated with HSU. Immigrants from some countries and some minority IMs possess special health beliefs and habits that can affect their HSU. A longer duration of residency would improve migrants’ social integration and familiarity with the healthcare system and decrease barriers to local medical insurance. Duration of residency in the destinations was found to be positively associated with HSU of immigrants (35, 89, 113, 114) and IMs (14, 15, 101, 115), while a few studies showed mixed results (87, 110).

Another factor associated with immigrants’ (64) and IMs’ (58, 103, 116) HSU is socioeconomic status. However, another article on IMs showed that this factor was not relevant (12). Socioeconomic status included different contexts that are related to living and working conditions. Some of these factors determine the most pressing problems that migrants suffer, which might include prior health problems. Referring to living conditions, studies on immigrants found that different types of accommodation (46, 113) and precarious living conditions (117) are associated with HSU. Working conditions associated with migrants’ HSU include work status and type of industry (for IMs), working conditions, employer-related factors, and job security. Employment status (100, 118, 119) and type of industry (11, 12, 55) were found to be associated with IMs’ HSU. Harsh working conditions are associated with immigrants’ HSU (43, 74). Unsupportive employers (82, 120), such as reluctance of employers to organize treatment for work-related accidents (45), would prevent immigrants from HSU. Barriers to immigrants’ HSU also included a lack of job security, such as fear of losing a job (63), a lack of discharge care (74), such as isolation, a lack of housing, food, and medical follow-up (74), and a loss of regular work wages (64, 74) or underpayment of medical leave wages (74). Some issues exist in both populations but are only described in one of these two populations. This difference might occur because of limited information.

In addition, literature on immigrants’ HSU showed that health belief identification (89) and health literacy (75, 98, 121) are associated with migrants’ HSU, and so are the IMs (58, 103). Quality of life (98) was also found to be a facilitator of immigrants’ HSU.

###### 3.2.3.1.2. Enabling resources

Enabling resources associated with HSU are constituted by social and financial resources and access to healthcare (24, 27). Incorporation of these necessary resources would directly facilitate the HSU.

Referring to social resources, social isolation or support was found to be associated with immigrants’ (71, 84, 98) and IMs’ (15, 47, 58, 122) HSU. Specifically, not having someone to trust and confide (89) and lacking a person to accompany the immigrants to the healthcare facilities (97) were found to be associated with a decrease in HSU by immigrants, while immigrants with more negative family relationships had more HSU (123) because of the poor health status. The number of friends (14, 55, 95) is positively associated with IMs’ HSU, while the number of family members yielded mixed results (17, 38).

Financial resources are another aspect of enabling resources associated with migrants’ HSU. First, financial constraints (120) are often measured by wage or income and reflect the purchasing capacity for commodities, including health services, thus affecting the HSU of both immigrants (76, 82, 89, 97, 124, 125) and IMs (14, 17, 50, 55, 70, 72, 94, 99, 126–128). Satisfaction with income (92) appears to be related to HSU. Some researchers indicated that the competing priorities of daily living were barriers to immigrants’ (121) and IMs’ (11) HSU. However, minimal research on IMs found financial constraints (12), and major financial sources (38) are irrelevant because they do not directly determine the purchasing capacity for healthcare services. Second, since participation in any medical insurance program could effectively reduce the medical burden, medical insurance (13, 95, 129) is another important aspect of financial resources associated with migrants’ HSU and whether insurance is associated with immigrants’ (77, 82, 88, 130–132) and IMs’ (11, 12, 50, 57, 72, 78, 92, 100, 127, 128, 133) HSU. Immigrants’ HSU is associated with paying the medical insurance premium regularly (114), which was associated with the availability of medical insurance.

The authors divided the access to healthcare (128) into time accessibility, geographic accessibility, and information accessibility. The former two factors would affect both immigrants’ and IMs’ (78) HSU. First, time pressures (79), including lack of leave for illness (63), scheduling conflicts (59, 69, 90), and time flexibility (97), are associated with immigrants’ HSU. Long working hours are associated with immigrants’ (45) and IMs’ (72) HSU. The number of rest days is also associated with immigrants’ HSU (125). Second, distance to the health facilities was also found to be associated with immigrants’ and IMs’ (58) HSU (40, 60, 64). Some indices are only described in immigrants because of limited information.

###### 3.2.3.1.3. Needs

Needs are another important individual-level factor that can be associated with migrants’ HSU (38, 64). Needs are measured by self-rated health and evaluated health assessments. A self-rated health assessment includes self-rated health and feeling different degrees of symptoms. In several studies, self-rated health was associated with immigrants’ (98, 114) and IMs’ (13, 17, 55) HSU. Feeling different degrees of symptoms was also found to be related to both immigrants’ (125) and IMs’ (50, 112, 126) HSU. Evaluated health can be measured by the disease status and the evaluated degree of symptoms. Disease status was associated with both immigrants’ (113, 131) and IMs’ (14, 17, 134) HSU. The evaluated degree of symptoms was related to IMs’ HSU in another study (135). Research on immigrants’ HSU found that reasons for hospitalization were not relevant (110), while the type of disease would determine IMs’ HSU (119).

##### 3.2.3.2. Subgroup-specific factors

Differences exist in terms of some specific variables of predisposing factors and enabling resources.

###### 3.2.3.2.1. Immigrant-specific factors

Referring to predisposing factors, some indices of demographic factors, including legal status and acculturation (language competence or communication barriers, discrimination or xenophobia, cultural difference, and self-identified), have only been associated with immigrants’ HSU. Legal status was an immigrant-specific factor, while reasons for migration have only been highlighted in studies concerning IMs. Specifically, immigrants with undocumented status (98, 136) had a lower level of utilization for various healthcare services (137) because their legal status was always a precondition for health system access. Acculturation is mentioned as a protective factor in the overwhelming majority of studies on immigrants’ HSU (138). These studies explain acculturation by language competence (40, 41, 43, 44, 46, 60, 62, 64, 65, 69, 76, 77, 79, 97, 114, 120, 124, 139, 140) or communication barriers (3, 117, 121), discrimination or xenophobia (3, 42, 43, 60, 65, 71, 120), cultural differences (62, 75, 76, 79, 89, 117, 121, 124), and self-identified (89). All factors induce poor affective comfort during the HSU process. Poor language competence and communication barriers also impede effective communication, which is very important to the health outcome and thus affects their HSU. Cultural differences would also affect communication (141).

Referring to enabling resources, information accessibility is a factor only frequently mentioned in immigrants’ HSU. Information accessibility or information barriers (42, 71, 117, 121) can be defined as a lack of awareness of laws and their rights regarding healthcare (62, 142, 143) and unfamiliarity with a new health system (41, 65, 77) (e.g., locations (82) and doctors (69)).

###### 3.2.3.2.2. IMs-specific factors

Migration range, reasons for migration, and acculturation (language) are associated with IMs’ HSU. Migration range (13, 14) was associated with IMs’ HSU because the cultural difference including health attitudes always increased but social resources decreased along with the migration distance. The reason for migration (14, 15) was found to be associated with IMs’ HSU because the need for health maintenance and economic status differed between IMs for different migration-related reasons. Language was also reported to be associated with IMs’ HSU (115) by acting as a measurement of social integration, except for some Indian IMs (144). Although language is associated with both immigrants’ and IMs’ HSU, the influencing mechanism of language is different. Immigrants’ language competence would determine their ability to accurately communicate with doctors, while the dialect competence of IMs would only affect their discrimination or xenophobia from local doctors.

Referring to enabling resources, characteristics of medical insurance are associated with the availability of the insurance at the selected destinations and thus are related to IMs’ HSU. For example, medical insurance type (14, 100, 145) and place of insurance enrollment (14, 17, 50, 56, 105) were found to be associated with IMs’ HSU. These features would determine the level of insurance compensation and flexibility for IMs to transfer their medical insurance between hometowns and destinations (16, 17). This is because some types of medical insurance are not available in the local health system, and these local characteristics would determine the availability of medical insurance.

##### 3.2.3.3. Mediating effects on the relationship between other determinants and HSU

Macro-structural/contextual factors and characteristics of healthcare delivery would affect the characteristics of the population at risk. First, non-health target policies could affect the characteristics of the population at risk. Limited by the policy, undocumented immigrants can seldom obtain work permits, which results in poverty and harsh living and working conditions (43). These precarious living conditions can also be associated with poor work-related housing (117). Second, healthcare policies affect the characteristics of the population at risk. For example, eligibility in enrollment of health insurance schemes, availability of cross-border health or social insurance schemes (26), and portability of health insurance in the hometown determine enrollment in available health insurance (134). Third, social, economic, and cultural environments can also influence migrants’ socioeconomic status, financial resources, social resources, and acculturation.

Characteristics of the healthcare delivery system can also affect the characteristics of the population at risk. The volume and organization can determine the access to healthcare of a country’s population, including migrants. Quality of service can affect the health outcome of migrants and their health service needs. The high costs of healthcare services have led to financial barriers to healthcare.

Finally, factors of the different dimensions of these characteristics of populations at risk are connected. On the one hand, factors affect the relationship between other factors and HSU. English language proficiency often mediates the relationship between gender and HSU (88). Social support has mediating effects on the relationships between HSU and independent variables, such as migrating duration and reason for migration (15), which also play a role in HSU. The unpredictable nature of out-of-pocket expenses can prevent migrants from seeking formal health services, thus ignoring their insurance enrollment (70). On the other hand, such factors can affect each other. Insurance enrollment was found to be associated with socioeconomic factors (116), including employment (11, 139), demographic characteristics (128), duration of migration (132), and financial constraints (70, 128). Language skills are often associated with the duration of migration (132). Time accessibility (70) and needs (133) can also be associated with financial constraints.

### 3.3. Choice of types/sites

Depending on the progression of the disease, the populations at risk can use different types of services, including preventive services (primary care), outpatient services/emergency services (secondary care), and inpatient services in sequence. The latter two are provided for patients, while the first was mainly provided for all of the populations. Emergency services are open almost all year round and can provide immediate help by specialists in the hospital (146), thus providing better access to healthcare services. Moreover, immigrant patients also use traditional services (147). Depending on the site of service providers, the patients can use services from pharmacies, clinics, and hospital services (including emergency services). The providers can be private or communal/public. Health providers in some countries can be divided into different levels with different levels of services (32), and patients can choose them freely (28–31).

The immigrants had a higher utilization rate of emergency services during off-peak hours (3, 90, 148, 149) and underuse of primary healthcare services (3, 147) and preventive services (147). As a result, immigrants had a higher risk of avoidable hospitalization (150). Referring to the site of healthcare, immigrants were found to be more likely to use healthcare services from public health facilities, while the general population also used private healthcare (151). However, IMs in some countries are more likely to visit private providers (33, 37), traditional healers (37), and pharmacies (72) because of the lower costs associated with these healthcare service formats. In countries with different levels of hospitals, IMs were found to be less likely to choose high-level hospitals than residents (33). The determinations could also be divided into three dimensions: (1) macro-structural/contextual factors; (2) characteristics of the health delivery system; and (3) characteristics of the population at risk. However, the influence mechanisms differed from those of HSU. Some determinants were also different from those of HSU.

#### 3.3.1. Macro-structural/contextual factors

Choice of types/sites was found to be associated with macro-structural/contextual factors, namely health policy, non-health targeted policies, and social, economic, and cultural conditions. The influence mechanisms were different from HSU choices. The healthcare policy regulates the rules of searching for healthcare, including the choice of sites. In some countries, patients, including migrants, can choose healthcare facilities freely (28–31), while in others, they can only utilize healthcare services in a certain order to obtain relatively cheap services (32, 152). The effects of non-health-targeted policies are also reflected in immigrants as obtaining the relevant documentation necessary to register their child’s birth is an important factor considered by immigrant women during their care-seeking decision-making (153), including the choice of sites.

Social conditions were also found to be associated with patients’ choices of sites. Insufficient research on migrants at present exists. IMs’ choice of sites for healthcare was found to be associated with the region (154) and province (22) in which they were living. In the general population, physical environment (155) is associated with patients’ choice of sites for health services, but the choice of cities was not relevant to their choices (156). Minority patients appear to be more likely to use services from hospitals with more minority patients (157).

#### 3.3.2. Characteristics of the health delivery system

Characteristics of a healthcare delivery system, namely volume, organization, quality of service, and cost, are also associated with the choice of healthcare type or site. These characteristics have different influence mechanisms on the HSU. Extensive research on the association between characteristics of the health delivery system and the general population’s choice of healthcare type or site has been done, while there was minimal research concerning immigrants and no research concerning IMs.

First, induced demand is a common problem in the health service market; thus, volume was found to be one important factor associated with the general population’s choice of healthcare site, but a lack of research on migrants exists. A higher density of hospitals was found to be associated with lower use of primary care facilities (158) among the general population. Higher physician densities are associated with a greater probability of visiting such health facilities (158, 159). However, insufficient research concerning migrants and the association between the volume and choice of sites is available. Second, previous studies had proved that organization (visit time or waiting time (146, 160, 161)) was an important factor associated with the choice of healthcare site with little research showing negative results (156) in the general population. In terms of immigrants, visit time and 24-h medical consultations (162) were found to be important factors associated with the choice of healthcare site because these factors reflect the efficiency of the health facilities and are associated with the time accessibility of immigrants. Third, motivated by quality preference, the quality of service (160, 163, 164) was also found to be an important factor associated with the general population’s choice of healthcare type and site. In this study, the quality of service was measured by different indices, such as facility size (160), reputation/word of mouth (155, 165), equipment (160, 166), providers’ medical skills (155, 160)/school of graduation (167) and/or the presence of a specialist (146, 166), perceived quality of care (164), providers’ interpersonal behavior (155), positive experience with the facility (146, 165, 166), specific quality information provided by performance reports (165), average health gain (168), and complication rates (169). In terms of immigrants, the quality of service (153) including providers’ medical skills (162, 170) was also reported to be associated with the choice of healthcare site. Fourth, in the perception of cost performance, cost (155), especially out-of-pocket cost (161) or affordability of a facility, was considered another factor associated with the choice of sites in the general population. Cost (153) was also found to be an important consideration for immigrants.

#### 3.3.3. Characteristics of the population at risk

The characteristics of the population at risk associated with the choice of sites also could be divided into predisposing factors, enabling resources, and needs. The internal mechanisms of these factors were also different from the HSU. Some issues exist in both populations but are only described in one of these two populations.

Predisposing factors were divided into demographic factors and socioeconomic status. Migrants with different demographic and socioeconomic characteristics reported different preferences as to the site or type of HSU. First, demographic factors were found to be associated with the choice of sites for IMs (22) and immigrants. Specifically, age (90, 162, 171), gender (90), and education (171–173) were reported to be associated with immigrants’ choice of sites and types (162, 171). IMs’ choice of healthcare site was also associated with education level (100). Furthermore, legal status, generation, race or ethnicity, migration characteristics, and acculturation were also found to be associated with the choice of healthcare site or type. Rural or urban registration (33) and the generation (50) were associated with the choice of healthcare site and type among IMs, and this finding might be associated with differences in their HSU habits. Similarly, nationality was also associated with the choice of healthcare type or site among immigrants (90, 173). Immigration background was not relevant in the choice of sites (174) in the general population in addition to IMs after considering the other confounding factors (175). Some variables are IM-specific factors. Migration characteristics (22), including migration range (176), were found to be associated with the choice of sites among IMs. Some variables are immigrant-specific factors. Not maintaining their identity was a major reason for the reluctance to seek treatment at a public hospital (172) among immigrants. Duration of migration (172) and language (153, 172) were also associated with the utilization of public hospitals among immigrants. Second, socioeconomic status was associated with IMs’ and immigrants’ choices of sites (22, 154). The type of industry was associated with obtaining medications from a drug store (172) among immigrants.

Enabling resources consisted of financial resources and access to healthcare. Better enabling resources for a healthcare facility was associated with higher utilization of this facility. First, financial resources included financial constraints and medical insurance. Financial constraints are often measured by wage or income and were associated with the choice of healthcare site or type (171) among IMs (127) and immigrants (171, 172). Medical insurance was another important aspect of financial resources associated with immigrants’ (172, 173) and IMs’ (127) choices of healthcare sites with some negative results (22, 154) among IMs. The average reimbursement rate of medical insurance (159) and/or out-of-pocket costs (161) were also found to be important considerations in the choice of healthcare site in the general population, but no study has been performed on migrants’ views of this aspect. Second, access to healthcare (170, 173), especially geographic accessibility (153), was associated with immigrants’ choice of healthcare site, but no study could be found concerning IMs. We did not find any research on the association between social resources and migrants’ choice of sites or types.

Needs were measured by self-reported health and evaluated health assessments. Self-rated health (173) was associated with immigrants’ choice of healthcare type. Feeling different degrees of symptoms was another factor considered by immigrants (74) and IMs (50) when choosing healthcare sites. Disease type was also associated with immigrants’ (173) and IMs’ (22) choice of healthcare site. They tended to choose higher-level health facilities, which are supposed to be of higher quality, for severe symptoms and diseases.

### 3.4. Medical locations

The choice of the location of healthcare services (named medical location) by the migrants could be divided into three categories: (1) hometown/home country, also named medical return; (2) host city/destination; and (3) other places, also named cross-border medical or medical tourism (20). The cost of healthcare might range across different regions within certain countries or different countries (21, 32) consistent with the level of economic development. The use of medical services in developed regions by populations from developing regions would help to meet their health need, attain the health system goals of developing countries (177), and stimulate improvements in the quality of local health services (178). Migrants’ preference for medical locations also depended on macro-structural/contextual factors, characteristics of the health delivery system, and characteristics of the population at risk. Some of the influence mechanisms were similar to the HSU and choice of types or sites; however, differences in the constituent and importance of determinants were found.

#### 3.4.1. Macro-structural/contextual factors

Macro-structural/contextual factors associated with medical locations consist of healthcare policy and social, economic, and cultural conditions. The influence mechanism differed from those of choice of types or sites. Limited by the healthcare policy, IMs in China could only get higher reimbursement from their medical insurance by returning for medical services before the promotion of the on-the-spot medical bill settlement system. Medical travel from Cambodia was driven and shaped by the interaction of the healthcare system and socio-economic or cultural factors at different levels, including regional trade liberalization and the pressure of relatives and other advisers in local communities (178). The medical diaspora organizations could facilitate health service utilization of immigrants in that particular destination (177), thus reducing their medical return. The locations in which the patients lived were also found to be associated with their medical travel (20).

#### 3.4.2. Characteristics of the health delivery system

The characteristics of the healthcare delivery system, including quality of service (178) and cost, were associated with patients’ medical locations. The influencing mechanism was similar to that of the choice of types or sites. However, the constituent of determinants was different. First, the pursuit of quality healthcare (179), perceived quality of healthcare (21, 180), and dissatisfaction with the current system (21, 181) would lead to an increase in transnational medical utilization of the immigrants. Perception of low quality of health services also was a motive for the medical return of immigrants (182–186). Second, cost (186, 187) or value for money (21) was found to be another factor associated with the medical location of immigrants. Some researchers indicated that affordability did not emerge as an independent motive but influenced the other factors (182). Moreover, seeking second opinions (21, 181, 182, 188) also emerged as a motivation for transnational medical utilization. No evidence of the relationship between medical locations and the volume or organization of the health delivery system was found.

#### 3.4.3. Characteristics of the population at risk

The characteristics of the population at risk included predisposing factors, enabling resources, and needs. Their influence mechanisms were similar to the HSU than the choice of types and sites. Some issues exist in both populations but are only described in one of these two populations.

Predisposing factors were divided into demographic factors and socioeconomic status. First, demographic factors, including gender, age, nationality, acculturation, duration in the destinations, and characteristics of migration, were associated with migrants’ medical locations. Acculturation was found to play a more important role in the choice of medical locations than the choice of types or sites. Gender (189), age (189, 190), and education level (174, 190) were associated with IMs’ medical locations. Nationality (181) was enumerated in immigrants’ medical returns. Social integration was associated with immigrants’ (188) and IMs’ (e.g., permanent settlement intention (18, 191)) medical locations. The immigrant population had a mixed result (192) in terms of social integration. Duration at the destinations was another factor associated with the healthcare locations of IMs (190). Some variables are IM-specific factors. The characteristics of migration, including the reason for migration (190) and migration accompanied by someone (191), were associated with IMs’ medical return. Some variables are immigrant-specific factors. Acculturation measured by language and communication (21, 180), discrimination experiences (188), and cultural differences (183, 193) was associated with immigrants’ medical locations for which the latter yielded mixed results (192). In addition, medical culture was previously mentioned in studies on immigrants (21, 181, 182, 187). Second, socioeconomic status, such as working status (118) and owning a house (190), was also associated with IMs’ medical return.

Enabling resources included financial and social resources and access to healthcare. These determinants and their influence mechanisms were found to be similar to the HSU. First, financial resources consisted of financial constriction and medical insurance. The financial resources had a mixed association with the healthcare locations (174, 194) in the general population. Although we did not find any literature on the association between migrants’ medical locations and financial constriction, financial status was a precondition for medical travel (183). Enrollment status in the accessible medical insurance was another factor associated with the medical locations of immigrants (21, 180, 184) and IMs (190, 191, 195), especially the local medical insurance (184, 191). The characteristics of medical insurance, such as the location/type (18, 195) and compensation rules (193) of a specific form of medical insurance, were also described in the literature concerning migrants. Second, social resources as measured by relatives living in their home country by immigrants (196) and family members living together by IMs (190) were found to be another factor associated with medical locations. Three, access to healthcare at the selected destination was also associated with migrants’ medical locations. Established healthcare records in the current residency (190) were found to be negatively associated with IMs’ medical return but having a usual source of care (180) was not a significant factor associated with immigrants’ medical return. The perception of availability (182, 185), including convenience (186) and time availability (21), was reported to be associated with immigrants’ medical returns. Furthermore, immigrants who had a high level of digital information technology use were more likely to undertake medical tourism than others with less technology use (192).

Needs measured by self-reported health and evaluated health were associated with immigrants’ (181) and IMs’ medical locations, but only research concerning immigrants was found. Self-reported health assessments were found to be associated with the medical return of immigrants (181, 184) with few negative results (180). Perceived severity of disease was also associated with immigrants’ medical return (77). Some disease types, such as major non-work-related injuries and chronic diseases (74), neoplasms, and diseases of the circulatory system (189), were associated with immigrants’ medical return (74) and populations’ medical travel (189).

## 4. Conclusion

Our study established a conceptual framework for migrant HSB. This conceptual framework included and delineated the determinants of immigrants’ and IMs’ HSB, provided new knowledge concerning the determinants of immigrants and IMs’ HSB, reclassified some dimensions of the determinants, explained the relationships between different characteristics of HSB, and indicated the differences in terms of their determinants. The results of this study may help facilitate a better understanding of the HSB of migrants and the management of medical insurance funds.

Following the previous models describing general populations and immigrants’ HSB, we divided the determinants of migrants’ HSB into three dimensions: (1) macro-structural/contextual factors; (2) characteristics of healthcare delivery system; and (3) characteristics of populations at risk. The classifications of determinants were consistent with Anderson’s model. The macro-structural/contextual factors consisted of healthcare policies, non-health-targeted policies, and specific regional context factors related to larger social, economic, and cultural conditions. Characteristics of the population at risk were categorized as predisposing factors, enabling resources, and needs. Characteristics of HSB were divided into different aspects, including site, type, location, and utilization. We also specified the mediating effects of some variables and the path of their influence on HSB.

We modified previous models. First, we distinguished the determinants of immigrants’ and IMs’ HSB. Some determinants, such as non-healthcare policies, the continuity of the healthcare system, legal status, acculturation, and information accessibility, were associated with immigrants’ HSB but seldom affected IMs’ HSB. Other factors associated with IMs’ HSB but ignored in the literature on immigrants included reasons for migration, migration range, and characteristics of the medical insurance. Determinants related to regional context were more complex among IMs, while only cultural aspects made sense for immigrants. Second, we reclassified some dimensions of the determinants. This new conceptual framework divides the characteristics of the health delivery system into volume, organization, quality, and cost. Access to healthcare was divided into time accessibility, geographic accessibility, and information accessibility. Information accessibility acted as an important barrier to immigrants’ HSU. Third, we divided the characteristics of HSB into site/type, locations, and utilization. We merged the type and site because they were found to be closely associated with each other and reflect healthcare accessibility and the quality of services the patients receive. Fourth, we highlighted the interaction of the site and location of HSB. By enumerating the determinants of site and location, respectively, we showed the difference in determinants between different aspects of HSB. However, this conceptual framework might need to be adjusted when studying the HSB of special populations, such as displaced persons. Moreover, the characteristic of time interval on HSB was not included in this conceptual framework. Revisions would be necessary if new determinants and relationships are found in future studies.

## Data availability statement

The original contributions presented in the study are included in the article/supplementary material, further inquiries can be directed to the corresponding author.

## Author contributions

BP developed the concept of the manuscript, conducted the literature search, developed the conceptual framework, and drafted the manuscript. LL conceptualized this study and manuscript. All authors provided critical feedback, revised the manuscript for intellectual content, and approved the final manuscript.

## Funding

This study was supported by the National Natural Science Foundation of China (Grant No. 71904211).

## Conflict of interest

The authors declare that the research was conducted in the absence of any commercial or financial relationships that could be construed as a potential conflict of interest.

## Publisher’s note

All claims expressed in this article are solely those of the authors and do not necessarily represent those of their affiliated organizations, or those of the publisher, the editors and the reviewers. Any product that may be evaluated in this article, or claim that may be made by its manufacturer, is not guaranteed or endorsed by the publisher.

## Abbreviations

HSB: health service behaviors
HSU: health service utilization
SDG: Sustainable Development Goal
IMs: internal migrants
